# Unsteady Aerodynamics of a Pitching Airfoil with Trailing-Edge Flap in a Four-Bladed Rotor Configuration

**DOI:** 10.3390/biomimetics11070498

**Published:** 2026-07-15

**Authors:** Dorin-Madalin Feraru, Daniel Măriuța, Teodor-Lucian Grigorie

**Affiliations:** 1 Faculty of Aerospace Engineering, National University of Science and Technology POLITEHNICA Bucharest, 060042 Bucharest, Romania; dorin.feraru@stud.aero.upb.ro; 2 Department of Aircraft Integrated Systems and Mechanics, Military Technical Academy “Ferdinand I” Bucharest, 050141 Bucharest, Romania; daniel.mariuta@mta.ro

**Keywords:** NACA 13112 airfoil, morphing airfoil, trailing edge flap, DSV, hysteresis

## Abstract

To improve the unsteady aerodynamic response of the IAR 330 PUMA rotor, the present analysis provides a two-dimensional (2D) CFD-based framework for rotor blade sections integrated with trailing-edge flaps (TEFs). From a biomimetic perspective, the TEF is treated as an engineering abstraction of the adaptive aft-chord and camber variation observed in natural flyers, providing a controlled morphing envelope for aerodynamic-load regulation. The scientific contribution consists of an integrated assessment of the NACA 13112 section over an extended TEF deflection range, the comparison of several relative TEF chord lengths, and the transfer of the section-level framework to a four-section representation of the IAR 330 PUMA rotor. First, the effect of TEF deflection on the trajectory and strength of the dynamic stall vortex (DSV) is examined for a pitching NACA 13112 airfoil with a chord length of c=0.6 m and a pitching axis located at x/c=0.25. The pitching motion was prescribed in ANSYS Fluent through a user-defined function (UDF), imposing a hysteresis variation of the angle of attack (AoA) from α=−3° to α=23°, while flap deflection angle (β) varied from β=−20° to β=8°, corresponding to upward and downward TEF deflection, respectively. The second part of this study extends the same pitching law to real-scale rotor blade sections under hovering flight conditions. For the rotor simulations, the Multiple Reference Frame (MRF) model was used for the steady-state analysis, whereas a Sliding Mesh interface was adopted for the transient computations. A 2D pressure-based solver was employed, together with the SST k-ω turbulence model, the Unsteady Reynolds-Averaged Navier–Stokes (URANS) formulation, and a coupled pressure–velocity scheme. The rotational speed was set to ω=265 RPM, corresponding to a local tangential velocity of approximately U=145 m/s at the analysed radius of r=5.225 m and to a local Mach number of M≈0.43. The ideal-gas assumption and energy equation were employed to account for compressibility effects. Among the investigated IAR 330 PUMA rotor-section configurations, the TEF with a chord length of $c_{f}=0.25c$ TEF provided the most balanced aerodynamic response, reducing the peak pitching-moment coefficient by approximately 32% relative to the baseline airfoil.

## 1. Introduction

**Figure 1:**
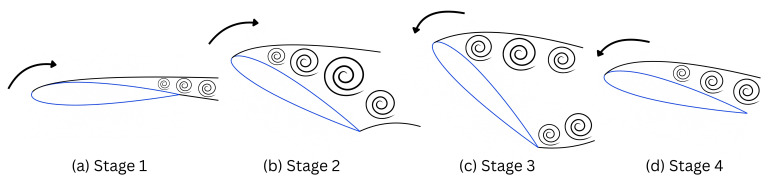
The stages of dynamic stall process: (**a**) static stall exceeded, flow reversal; (**b**) DSV convection, lift increase; (**c**) lift stall; full separation; (**d**) flow reattachment.

Dynamic stall is a time-dependent flow process associated with airfoils subjected to rapid variations in AoA, where stall onset is postponed beyond the corresponding static stall limit (see Figure 1). The resulting flow field differs significantly from that of static stall, as it is dominated by transient separation processes, the formation and convection of a DSV, and rapid variations in aerodynamic loads. Such effects are commonly observed on turbomachinery cascades, vertical-axis wind turbines (VAWTs), manoeuvring aircraft, and helicopter rotor blades, where they may enhance or degrade aerodynamic performance depending on the specific flow regime [1,2,3].

For standard rotorcraft configuration under forward-flight conditions, the advancing-side blade operates at a lower AoA than the retreating-side blade because its higher local relative velocity helps preserve rotor lift balance [4]. At high advance ratios and large thrust coefficients, the AoA on the retreating blade may exceed the corresponding static stall limit, thereby promoting the onset of dynamic stall. This phenomenon can generate excessive loads in the blade pitch-link system and may ultimately restrict the operational flight envelope of the helicopter [5]. From a flow-physics perspective, the DSV governs the transient aerodynamic response of the retreating blade. Its downstream convection delays massive flow separation and induces a temporary lift overshoot, followed by an abrupt pitching-moment variation as the vortex approaches the trailing edge [6].

Dynamic stall was investigated primarily through experimental methods until the mid-twentieth century, as the limited theoretical and empirical knowledge available at that time constrained the development of reliable mathematical models for describing its unsteady flow mechanisms. Friedmann contributed significantly to the early theoretical interpretation of dynamic stall by outlining three representative modelling frameworks in the late 1970s [7,8]. These modelling approaches progressively evolved from relatively simplified formulations to more advanced dynamic stall models, including the Boeing, ONERA, Leishman–Beddoes, and Johnson models [8,9,10].

McCroskey et al. [11,12] established one of the earliest systematic experimental frameworks for interpreting dynamic stall by performing oscillating-airfoil investigations. Rather than treating the phenomenon as a single aerodynamic event, their analysis differentiated the flow response according to the maximum AoA attained during the pitching cycle. This led to the identification of four distinct stall regimes, namely, unstalled regime, incipient stall regime, mild stall regime, and fully developed stall regime. A more refined characterization of dynamic stall became possible through time-resolved particle image velocimetry (TR-PIV), which allowed Mulleners and Raffel [13,14] to decompose the flow evolution into five successive stages and to identify the primary and secondary instability mechanisms involved in stall development.

The numerical investigation of dynamic stall was initially advanced through laminar-flow simulations based on the complete Unsteady Navier–Stokes equations, with Mehta reporting one of the first computations for an oscillating airfoil [15]. However, at high Reynolds numbers, the turbulent characteristics of the flow field require the adoption of appropriate turbulence-closure models in order to resolve the complex separation and vortex-dominated mechanisms associated with dynamic stall [16].

TEF actuation is regarded as a promising active-control strategy for mitigating the adverse aerodynamic effects associated with dynamic stall [17]. Feszty et al. [18] attributed the loss of aerodynamic damping and the pronounced negative pitching-moment response during DSV development primarily to the aft-chord vortex structure. Gerontakos and Lee [19] further examined TEF deflection on a dynamically pitching airfoil by evolving the flap-deflection duration, onset time, and amplitude. Their wind-tunnel results showed that, although TEF actuation did not significantly modify DSV formation or detachment, variations in maximum lift were primarily associated with the duration of flap deflection. Krzysiak and Narkiewicz [20] also demonstrated that an increase in the maximum lift coefficient can be achieved through TEF oscillations performed at different frequencies when flap deflection and the airfoil AoA grow simultaneously and the phase relationship between both motions is properly considered.

Recent studies have advanced the understanding and control of dynamic stall through numerical modelling and adaptive geometry. Xing et al. [21] examined the influence of TEF motion on DSV evolution and unsteady aerodynamic loads, while Wu et al. [22] showed that phase-shifted trailing-edge morphing modifies the separated shear layer, coherent-vortex development, and wake structure around a pitching airfoil. Sterpu et al. [23] demonstrated the suitability of UDF-prescribed periodic motion for reproducible dynamic-stall simulations. At rotor-section level, Liang et al. [24] and Kargarian and Karimian [25] further confirmed the relevance of active geometric adaptation for unsteady load alleviation. Together, these studies provide the direct context for the present NACA 13112 TEF investigation.

From a biomimetic standpoint, the TEF considered in this study is not intended to reproduce a specific biological structure, but rather to abstract a functional principle commonly observed in natural flyers: the active modification of trailing-edge geometry to regulate aerodynamic loading under unsteady flow conditions. Birds, bats, and insects can alter local camber, surface curvature, and aft-chord shape during manoeuvres, gust encounters, or high-AoA conditions, thereby redistributing pressure, influencing separation development, and controlling vortex formation [26,27,28]. In the present work, this biological strategy is translated into a simplified engineering model through a morphing trailing-edge region with prescribed β and chordwise extent. The investigated TEF chord lengths, $c_{f}=0.15c$, $c_{f}=0.25c$, and $c_{f}=0.35c$, together with the deflection interval β=−20° to β=8°, are interpreted as an engineering morphing envelope rather than as a direct quantitative reproduction of a specific bird or bat wing. These values were selected to examine the progressive influence of aft-chord control authority and the compromise between lift retention, drag and pitching-moment alleviation [29]. By combining the TEF deformation with a prescribed pitching motion, the simulations examine how bio-inspired chordwise shape variation modifies pressure redistribution, dynamic-stall vortex evolution, hysteresis behaviour, and unsteady aerodynamic loads on both the pitching airfoil and the rotor blade sections. In this way, this study uses the TEF as a bio-inspired flow-management concept for assessing how adaptive trailing-edge morphing may support lift control and load alleviation in rotorcraft applications.

**Figure 2:**
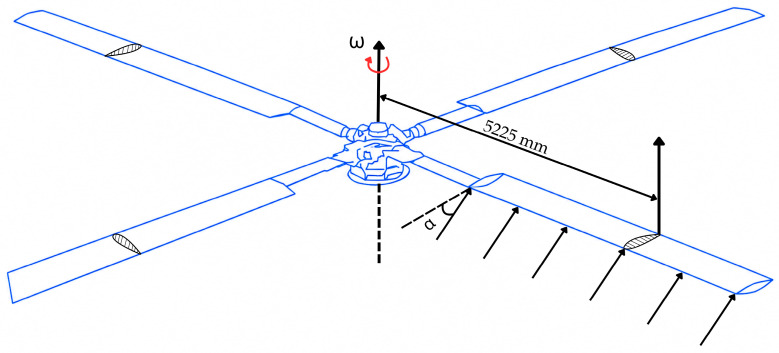
Blade sections of the IAR 330 PUMA rotor considered in the numerical simulations [30].

The present study investigates the unsteady aerodynamic response of a pitching NACA 13112 airfoil equipped with a TEF using an established two-equation URANS framework implemented in ANSYS Fluent 2022 R1 (see Figure 2). Particular attention is devoted to the use of a UDF for prescribing the airfoil pitching motion and to the capability of the numerical approach to characterize the principal unsteady flow mechanisms associated with dynamic stall. The analysis focuses on DSV generation, convection, and intensification, wake development during the pitching cycle, and the corresponding variations in the unsteady aerodynamic loads. In addition, the influence of TEF deflection on the aerodynamic response is examined. To extend the relevance of the investigation from the pitching-airfoil configuration to a rotorcraft application, the same numerical framework is further applied to four NACA 13112 blade sections representative of the IAR 330 PUMA rotor. The contribution of this study lies in the integrated aerodynamic assessment of several β and $c_{f}$ configurations and in the transfer of the section-level analysis to a four-section rotor representation. The NACA 13112 section also provides the geometric basis for subsequent three-dimensional (3D) blade-section studies addressing actuator integration, the available internal volume, the hinge and transmission arrangement, and the required local structural reinforcement.

## 2. Details of the (TEF)/Kinematic Definition

### 2.1. Airfoil Geometry

The numerical simulations were conducted using the NACA 13112 airfoil, defined with an airfoil chord of c=0.6 m. Prior to the onset of the prescribed motion, the airfoil was positioned at AoA=0°, which served as the reference state for the subsequent dynamic-stall analysis. The unsteady pitching motion was imposed about the aerodynamic reference axis, located at x/c=0.25 on the airfoil camber line. Within the UDF implementation, the quarter-chord location was specified as the center of gravity (CG), thereby defining the rotational reference point about which the prescribed pitching motion of the airfoil was imposed in ANSYS Fluent 2022 R1.

### 2.2. TEF Geometry ($c_{f}$, TEF Hinge Axis, β)

**Figure 3:**
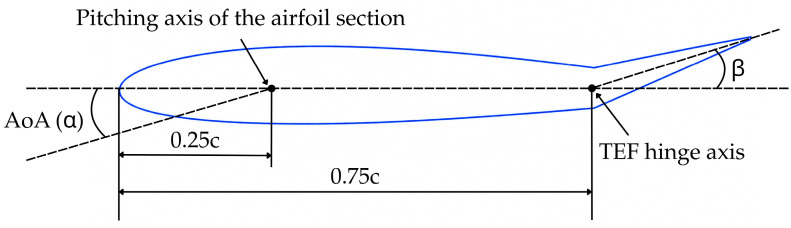
Conceptual schematic illustrating the prescribed rotational kinematics of the TEF.

The single-axis hinge-based articulation and deflection kinematics of the TEF examined in the present investigation are schematically represented in Figure 3. The TEF chord fraction relative to the main airfoil chord is established at $c_{f}=0.25c$. A negative deflection angle β corresponds to an upward displacement of the flap surface. The geometric configurations of the airfoil section under varying deflection conditions are illustrated in Table 1. Within the computational framework, upward rotation of the TEF causes the trailing-edge terminus to pivot about the designated hinge axis, while the adjacent upper and lower flap surfaces undergo proportional geometric scaling.

### 2.3. Pitching Motion Law

**Figure 4:**
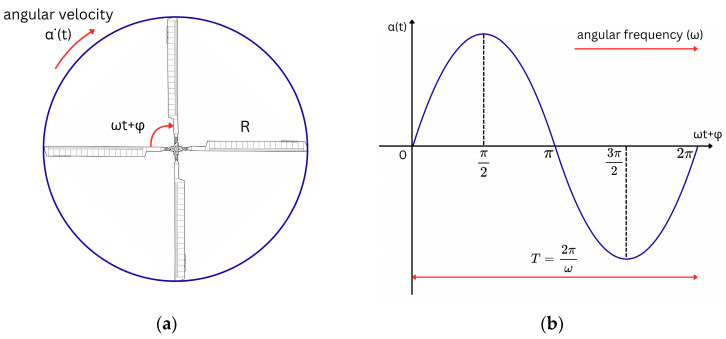
Airfoil pitching-law schematic: (**a**) circular motion; (**b**) harmonic response.

The prescribed motion of the pitching airfoil was implemented in ANSYS Fluent 2022 R1 through a UDF, developed as a compiled C-language subroutine coupled with the solver. In the adopted formulation, the airfoil kinematics were governed by a time-dependent sinusoidal pitching law, through which the instantaneous AoA varied periodically about a mean AoA within predefined angular limits. This mathematical description ensured a continuous and repeatable evolution of the airfoil AoA during the unsteady cycle, providing the required kinematic framework for reproducing the aerodynamic response associated with dynamic-stall development (see Figure 4).

The instantaneous AoA was defined as follows:

(1) $$\alpha \left(t\right)=\;a_{0}+A_{\alpha }sin\left(\omega t+\varphi \right),$$

where $\alpha_{0}$ is the mean AoA, $A_{\alpha }$ represents the pitching amplitude, ω is the angular frequency of the imposed oscillation, and φ is the initial angular offset. The mean AoA and pitching amplitude were defined from the prescribed minimum and maximum AoA limits as follows:

(2) $$\alpha_{0}\;=\;\frac{\alpha_{max}+\alpha_{min}}{2},$$

(3) $$A_{\alpha }=\frac{\alpha_{max}-\alpha_{min}}{2}.$$

Depending on the investigated numerical configuration, the angular frequency of the imposed motion was defined either through the reduced-frequency formulation or from the rotor angular speed. Thus, for a pitching airfoil case, the angular frequency may be related to the frequency parameter, far-field velocity, and airfoil chord as follows:

(4) $$\omega =\;\frac{2kV_{\infty }}{c},$$

where k is the frequency parameter, $V_{\infty }$ is the far-field velocity, and c is the airfoil reference chord. For rotor-based configurations, the imposed angular frequency may instead be associated with the rotor rotational speed, according to

(5) $$\Omega =\frac{2\pi RPM}{60}.$$

(6) $$\dot{\alpha }\left(t\right)=A_{\alpha }\omega \cos\left(\omega t+\varphi \right).$$

### 2.4. IAR 330 PUMA Rotor Aerodynamics

The mechanical power associated with the rotor operation, denoted by *P*, is obtained from the total aerodynamic torque *M* and the rotor angular velocity ω:

(7) P=Mω.

**Figure 5:**
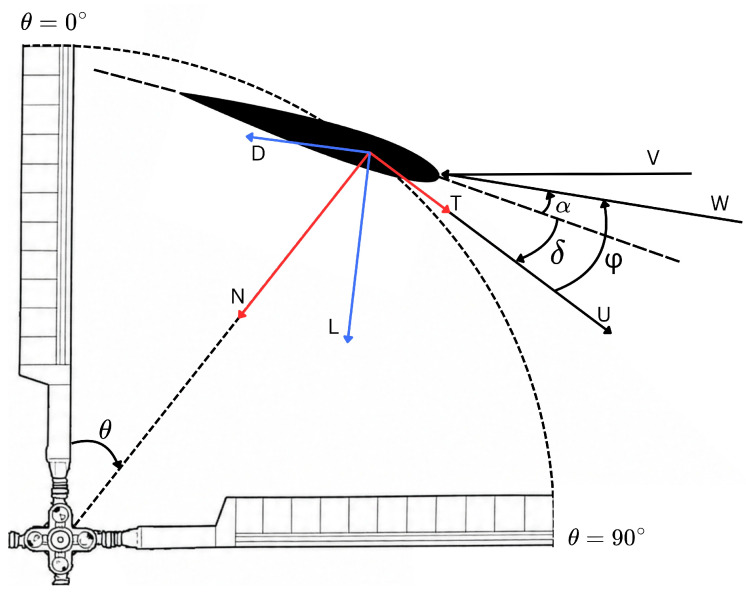
Velocity and aerodynamic force vectors acting on the rotor blade section, defined with respect to the azimuth angle θ and the local relative flow velocity W.

The velocity vectors and aerodynamic forces acting upon an individual blade element are illustrated in Figure 5. The total torque can be computed by considering the tangential force component *T*:

(8) $$M=n_{b}\bar{T}r,$$

where $n_{b}$ indicates the number of rotor blades, $\bar{T}$ represents the mean tangential force component acting on each blade, and r denotes the radial position of the investigated blade section, measured from the rotor hub center.

The relative flow velocity results from the combination of the rotational blade velocity and the local inflow velocity at the rotor disk. The aerodynamic load is decomposed into lift and drag components and subsequently projected into tangential and normal directions relative to the rotor path. For a rotor blade section positioned at a spanwise position r from the rotor hub, the circumferential velocity induced by rotor rotation is

(9) $$U=\omega r\hat{\theta },$$

where $\hat{\theta }$ denotes the circumferential unit vector associated with the positive sense of rotor rotation. The relative wind velocity *W* comprises two constituent components: the local velocity across the rotor disk *V*, with the blade-section velocity *U* resulting from rotor rotation:

(10) W=V−U.

The magnitude of the relative velocity can be determined through

(11) $$\left|W\right|=V\sqrt{{\left(\frac{\omega r}{V}+\sin\theta \right)}^{2}+{\left(\cos\theta \right)}^{2}.}$$

The inflow angle φ, defined by the orientation of the relative velocity vector W, is

(12) $$\varphi =\tan^{-1}\left(\frac{\cos\theta }{\frac{\omega r}{V}+\sin\theta }\right).$$

The instantaneous AoA, α, is established from the geometric relationship between the relative-wind orientation, φ, and the imposed blade pitch angle, δ, as follows:

(13) α=φ+δ.

The local speed ratio, λ, a fundamental non-dimensional parameter characterizing rotor operational state, is expressed as follows:

(14) $$\lambda =\frac{\omega r}{U}.$$

The aerodynamic load acting on the blade section is decomposed into normal and tangential force components, denoted by N and T, respectively:

(15) N= Lcosφ+Dsinφ,

(16) T=Lsinφ−Dcosφ,

where L and D denote the lift and drag forces generated by the blade section, respectively, and are determined from the aerodynamic coefficients according to:

(17) $$L=\frac{1}{2}\rho W^{2}A_{blade}C_{l},$$

(18) $$D=\frac{1}{2}\rho W^{2}A_{blade}C_{d}.$$

$C_{l}$ is associated with lift generation, whereas $C_{d}$ characterizes the drag contribution, while the reference area $A_{blade}$ is defined for a unit span (*S*) of the analysed blade section. Based on the aerodynamic moment coefficient, $C_{m}$, the power coefficient ($C_{P})$ can be determined by relating the rotor torque to the corresponding rotational power:

(19) $$C_{P}=\frac{P}{\frac{1}{2}\rho U^{3}A_{blade}},$$

(20) $$C_{m}=\frac{M}{\frac{1}{2}\rho U^{2}A_{blade}r},$$

(21) $$C_{P}=C_{m}\lambda .$$

Equations (8)–(18) are adapted from Dyachuk [31], whereas Equations (19)–(21) follow Mathew [32]. Since the rotor operates in hover, the representative velocity was defined as the local tangential velocity of the analysed blade section. At r=5.225 m, this velocity is approximately U=145 m/s, yielding

(22) λ≈1,

(23) $$C_{P}\approx C_{m}.$$

## 3. Numerical Methodology

The present numerical methodology adopts a 2D section-level formulation to isolate the influence of TEF geometry and prescribed pitching kinematics on the dominant aerodynamic mechanisms associated with dynamic stall. All investigated configurations are analysed under identical boundary conditions, temporal discretization, turbulence treatment, and motion laws, thereby enabling the effects of β and $c_{f}$ to be assessed without the additional variability introduced by blade twist, taper, radial flow, tip-vortex development, and structural deformation. At the selected radial station, the model resolves the principal chordwise processes governing the unsteady response, including leading-edge shear-layer separation, DSV formation and downstream convection, pressure-centre migration, aft-chord pressure recovery, aerodynamic hysteresis, and wake recovery. The prescribed motion is imposed through a UDF, ensuring a repeatable AoA history and a phase-consistent comparison of the aerodynamic coefficients and flow structures. The resulting data are not interpreted as a complete 3D rotor prediction, but as section-level aerodynamic trends and load envelopes for subsequent 3D aerodynamic, structural, and actuator-integration studies.

### 3.1. Part I—Pitching Airfoil Study

In its present implementation, the UDF reproduces a complete dynamic stall cycle, with the AoA varying from α=−3° to α=23° and returning to α=−3°. This interval was selected in accordance with the operational documentation of the IAR 330 PUMA helicopter, in order to investigate the transitional regime in which dynamic stall effects become significant. The pitching law enables a controlled and reproducible description of the unsteady airfoil motion, making the model suitable for assessing different airfoil configurations under dynamic stall conditions. The resulting flow field was analysed with particular attention to the onset of dynamic stall, the configuration of TEF-morphed airfoils, and the downstream evolution of the wake at multiple chord-based distances.

**Figure 6:**
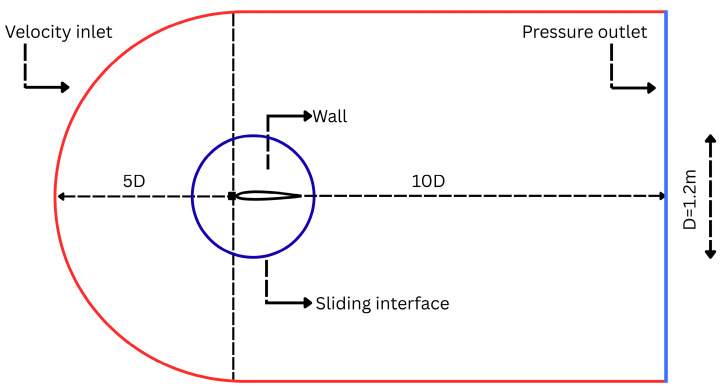
C-shaped transient computational domain used for the NACA 13112 airfoil simulations.

A C-shaped transient computational domain was constructed around the NACA 13112 airfoil, as illustrated in Figure 6. The airfoil chord was set to c = 0.6 m, while a unit span of S = 1 m was assumed for the 2D formulation. A prescribed-velocity condition was imposed at the upstream boundary, whereas the outflow boundary was defined as a pressure outlet, ensuring an appropriate representation of the external flow through the domain. The far-field extension was set to 5D upstream and 10D downstream in order to reduce boundary-induced effects on the unsteady aerodynamic response. The domain was divided into a stationary outer region and an oscillating inner region, coupled through a circular sliding interface of radius R = 0.6 m, with its centre located at the pitch-axis position x/c = 0.25. The airfoil surface was treated as a no-slip wall, while translational periodic boundary conditions were imposed on the spanwise faces.

Grid sensitivity was assessed with coarse, medium, and fine meshes while the geometry, pitching kinematics, time-step size, turbulence model, and discretization schemes were kept unchanged. Only the prescribed spatial-resolution controls were modified, allowing the effect of grid density on the unsteady aerodynamic loads to be isolated. The refinement procedure follows the coordinated approach reported by Xing et al. [21], in which the surface spacing, first-layer height ($h_{1}$), and inflation growth rate are varied consistently. The resulting mesh parameters are summarized in Table 2.

The grid sequence provides a progressive refinement of the wall-adjacent region, airfoil surface, near wake, and outer flow field. Relative to the coarse grid, the medium mesh increases the cell count by a factor of 2.64, whereas the transition from the medium to the fine grid requires a further factor of 2.01. At the same time, the reduced surface spacing and $h_{1}$, together with the larger number of inflation layers and lower growth rate, produce a smoother wall-normal expansion and improve the resolution of the separated shear layer. This coordinated strategy avoids refining a single region in isolation and provides a consistent basis for evaluating the influence of spatial discretization on the dynamic-stall response.

**Figure 7:**
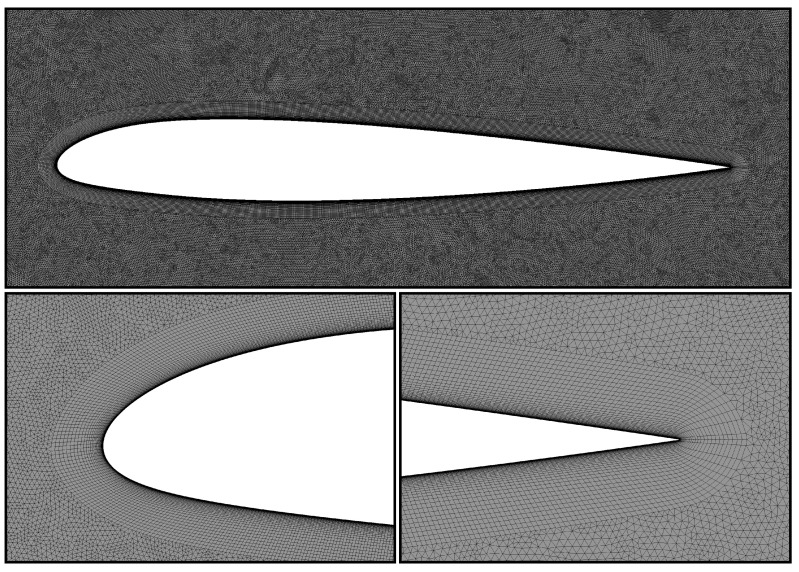
Computational mesh for the transient NACA 13112 pitching airfoil simulation, showing the global topology and the refined leading edge, trailing edge, and near wall regions.

The comparison showed that the principal change occurs between the coarse and medium solutions, whereas the fine grid mainly adds local resolution at substantially higher computational cost. The medium mesh was therefore selected as the reference grid for the subsequent NACA 13112 simulations. It employs $N_{infl}\;=\;50$ inflation layers and a first-layer height of $h_{1}=3.20\;\times \;10^{-3}\;\mathrm{mm}$, with additional refinement near the leading edge, trailing edge, and near wake. Figure 7 illustrates the resulting topology and the gradual transition from the wall-adjacent layers to the surrounding unstructured region.

The numerical methodology was also evaluated against the wind-tunnel measurements reported by McAlister et al. [11] for the Sikorsky SC-1095 airfoil. These data serve as a method-level validation of the dynamic-stall modelling approach and are not presented as experimental results for the NACA 13112 section. The SC-1095 measurements cover an AoA interval of approximately $\alpha =\left[0{}^{\circ}\text{–}18{}^{\circ}\right]$, whereas the present NACA 13112 calculations extend from α=−3° to α=23°.

Because the airfoil geometry and pitching interval are different, exact point-by-point agreement in the load extrema or hysteresis-loop width is not expected. The relevant validation criterion is whether the CFD framework reproduces the characteristic dynamic-stall sequence: delayed loading during the upstroke, the orientation of the hysteresis loops, the post-stall drag rise, and the negative pitching-moment drop associated with DSV convection. This comparison therefore establishes the physical credibility of the numerical method before its application to the NACA 13112 configuration.

**Figure 8:**
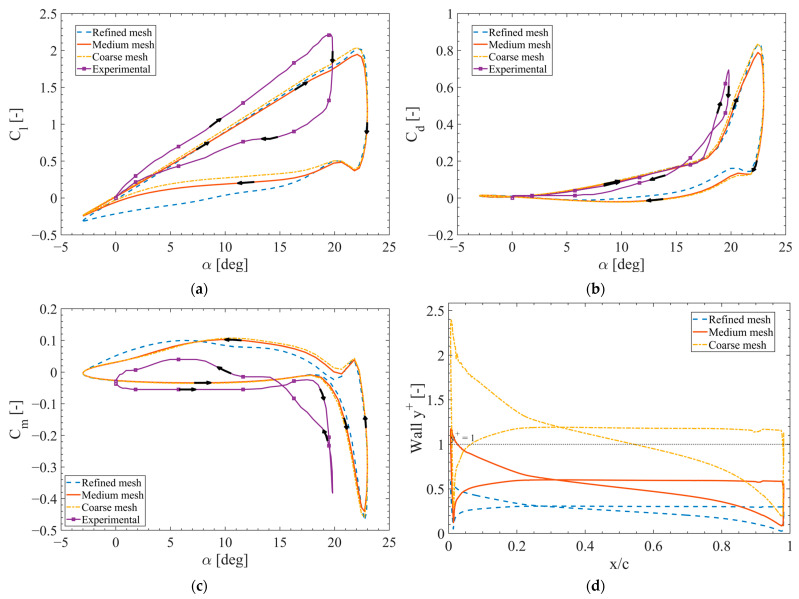
Grid-sensitivity assessment for the pitching NACA 13112 airfoil, with arrows indicating the upstroke and downstroke directions, and method-level comparison with the SC-1095 wind-tunnel data reported by McAlister et al. [11]: (**a**) variation of $C_{l}$; (**b**) variation of $C_{d}$; (**c**) variation of $C_{m}$; (**d**) wall $y^{+}$ distribution.

Figure 8d shows that the medium and fine grids maintain $y^{+}$ close to or below unity over most of the chord, whereas the coarse grid reaches a localized maximum of approximately $y^{+}=\left[2\text{–}2.5\right]$ near the leading edge. The small medium-to-fine change confirms that the reference mesh adequately resolves the viscous sublayer for the SST k–ω formulation. In Figure 8a, the medium and fine $C_{l}$ loops remain nearly coincident throughout the attached-flow region, with differences generally below approximately 2–3% before stall. The coarse solution shows the largest departure during the rapid load increase and around the loop closure, indicating greater sensitivity to surface and wake resolution. The SC-1095 experimental curve is considered only in terms of its general hysteresis orientation and delayed-stall signature; its peak values are not used to quantify the NACA 13112 grid error.

The drag and pitching-moment histories provide a more demanding test in the deep-stall portion of the cycle. For $C_{d}$, the medium and fine curves reproduce almost the same onset and slope of the post-stall rise, while the coarse grid exhibits a slightly broader transition and a larger local separation between the upstroke and downstroke branches. A similar result is obtained for $C_{m}$: the medium and fine grids predict nearly the same phase and magnitude of the sharp negative drop, whereas the coarse mesh produces the most visible modification around the minimum and recovery portions of the loop. The experimental SC-1095 curves display the same qualitative drag increase and negative moment response, supporting the physical sequence reproduced by the CFD model without implying direct quantitative agreement between the two airfoils.

Taken together, the coefficient histories show that the dominant grid effect occurs between the coarse and medium solutions. Further refinement to the fine mesh produces only limited changes in the attached-flow branches, the onset of the load excursions, and the phase of the DSV-induced peaks. This convergence, combined with the near-wall response in Figure 8d, indicates that the medium grid captures the principal unsteady mechanisms required for the present analysis. The fine mesh mainly increases local spatial resolution at a substantially higher computational cost, without materially changing the aerodynamic trends. The medium mesh was therefore retained for the subsequent NACA 13112 simulations as the most appropriate compromise between numerical accuracy and computational efficiency.

### 3.2. Part II—Four-Blade Rotor with TEF

The rotor analysis extends the section-level methodology to four NACA 13112 sections positioned at successive azimuthal locations. This arrangement enables the aerodynamic response of the individual sections and the resulting combined rotor loading to be examined within the same numerical framework, while preserving a direct comparison with the pitching-airfoil results. The configuration is therefore used to evaluate the phase-dependent influence of TEF actuation on sectional coefficients, rotor moment, and wake development under hovering conditions.

**Figure 9:**
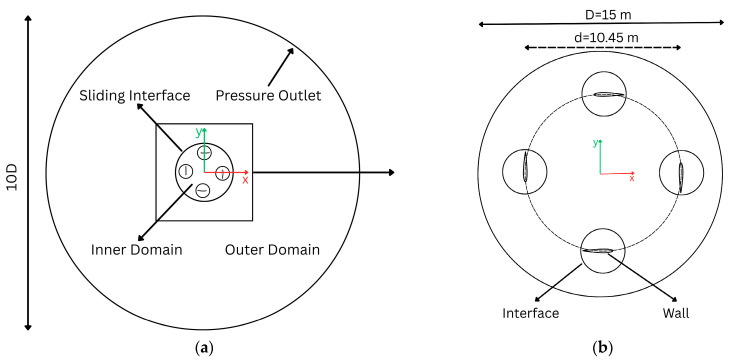
Numerical flow region for the rotor simulations: (**a**) global domain layout; (**b**) rotating region with blade sections and sliding interfaces.

The analysed rotor configuration is derived from representative full-scale helicopter blade sections based on the NACA 13112 airfoil, selected for its suitability in further investigations involving a TEF actuation mechanism. The principal geometric parameters of the analysed rotor sections are summarized in Figure 9, while the complete rotor layout, operating conditions, and performance-related data are provided in [33]. Since helicopter rotors operate under open-flow conditions, the computational domain must be sufficiently extended to reduce blockage effects and artificial boundary interference [34,35]. Mohamed et al. [36] showed that insufficient far-field clearance may significantly affect rotor performance coefficients. Therefore, the domain boundaries should be positioned at a distance equivalent to at least 10D from the rotor hub to minimize boundary-induced effects.

The rotor computational runs were performed using a domain composed of a rotating inner region and a stationary outer region. The fixed region represents the global control volume, while the rotating region contains the rotor blade sections and allows the angular motion to be imposed through the Sliding Mesh approach. In this study, the stationary domain was defined by a diameter of D=150 m, whereas the rotating region had a diameter of d=15 m. The rotating region was divided into four separate sub-domains, each enclosing one blade section, in order to enable the application of the Unsteady Sliding Mesh model.

For the pitching NACA 13112 airfoil case, a C-grid mesh was adopted to ensure a smooth discretization of the external flow field and an adequate resolution of the wake region. For the rotor configuration, O-type grids were generated around each blade section, enabling the near-wall cells to conform to the airfoil curvature and to provide consistent refinement along the suction side and around the leading-edge and trailing-edge regions. As in the pitching-airfoil setup, the rotor mesh was refined close to the blade surfaces to maintain wall $y^{+}$ values close to unity, ensuring proper resolution of the viscous sublayer during the transient simulations.

A hybrid meshing strategy was employed, combining structured inflation layers near the blade walls with unstructured elements in the remaining flow field. Additional refinement was introduced near the blade surfaces and downstream of the trailing edges, where separation, vortex formation, and wake interaction are expected to occur. This mesh topology provides sufficient resolution of the main unsteady aerodynamic mechanisms while preserving numerical efficiency for the complete rotor simulation [37,38,39,40].

A three-level mesh-sensitivity analysis was performed for the four-blade rotor configuration using coarse, medium, and fine grids (see Table 3). The refinement strategy was applied consistently across the stationary and rotating domains, the local blade-section regions, the airfoil surfaces, and the near-wall inflation layers, while all physical and numerical settings were kept unchanged. The coarse grid exhibited a 23.69% deviation in $C_{P}$ relative to the fine-grid solution, whereas the medium grid reduced this difference to 5.72%, indicating a substantial improvement in solution stability after the first refinement step. At the same time, the maximum and average skewness values remained within an acceptable range for all three meshes, confirming that the refinement process increased spatial resolution without compromising element quality. The transition from the medium to the fine grid required an approximately 81% increase in cell count, while producing only a comparatively small change in the global power response, thereby indicating a clear reduction in grid sensitivity.

**Table 3:** Mesh-refinement and $C_{P}$ sensitivity for the four-blade rotor configuration.

Mesh Control	Mesh Parameter	Coarse Mesh	Medium Mesh	Fine Mesh
Body Sizing	Rotating domain element size [mm]	40	25	20
	Stationary domain element size [mm]	350	250	200
	Airfoil domain [mm]	8	5	3
Edge Sizing	Airfoil edge size [mm]	4	2.5	2
Inflation	First-layer height [mm]	$6.40\times 10^{-3}$	$3.20\times 10^{-3}$	$3.20\times 10^{-3}$
	Number of inflation layers	45	65	65
	Inflation growth rate	1.15	1.10	1.1
Elements Number	Total number of cells	$7.51\times 10^{5}$	$1.69\times 10^{6}$	$3.06\times 10^{6}$
Mesh Quality	Maximum skewness	0.71669	0.71756	0.75502
	Average skewness	$6.30\times 10^{-2}$	$6.20\times 10^{-2}$	$6.10\times 10^{-2}$
$C_{P}$	Difference of $C_{P}$ with respect to the fine mesh [%]	23.69%	5.72%	/

**Figure 10:**
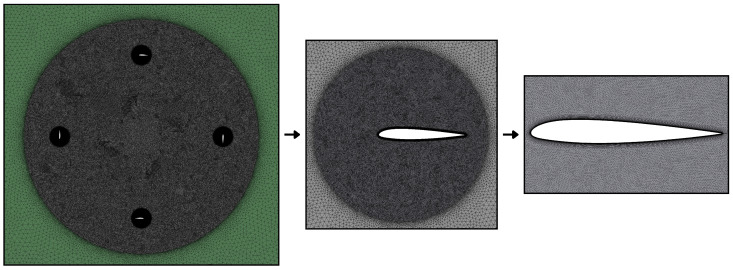
Hybrid mesh topology used for the rotor configuration, with structured near-wall grids and unstructured elements in the outer domain.

Based on these results, the medium mesh was selected for the subsequent rotor simulations as the most appropriate compromise between numerical accuracy and computational cost (see Figure 10). The remaining difference relative to the fine grid is considerably smaller than the coarse-to-medium variation, showing that the dominant grid-related changes are already captured at the medium resolution. The selected topology preserves sufficient refinement around all four blade sections, across the sliding interfaces, and within the near-wake regions, where separation, vortex convection, and blade-to-blade aerodynamic interaction are expected to be most sensitive to spatial discretization. In addition, the near-wall treatment remains adequate for resolving the boundary-layer development and the onset of separation, while the gradual transition toward the unstructured rotating and stationary domains limits artificial numerical diffusion.

The medium mesh therefore retains the principal flow structures and global loading trends required for the transient rotor analysis, while avoiding the substantially higher computational cost associated with the fine grid [37,38,39,40].

### 3.3. Numerical Setup and Transient Simulation Parameters

The numerical computations were conducted within the ANSYS Fluent 2022 R1 using a URANS formulation. The solver configuration was initially established for the pitching-airfoil case and subsequently adapted for the rotor simulations. Owing to the relative velocity at the investigated blade section, approximately V=145 m/s, corresponding to M≈0.43, the energy equation was activated, and air density was described using the ideal-gas law, while the dynamic viscosity was prescribed as $\mu =1.7894\times 10^{-5}\;\mathrm{kg}/\left(m\cdot s\right)$. Although the flow regime remains subsonic, the selected formulation allows compressibility effects to be considered during the transient analysis.

For the pitching-airfoil simulation, the prescribed motion was advanced with a time-step size of Δt=0.00126 s, using $N_{steps}=360$ physical time steps and a maximum of $N_{iter}=20$ inner iterations per time step. The airfoil completed $N_{rev}=5$ full pitching cycles between α=−3° and α=23°, with the final stabilized cycle retained for the aerodynamic hysteresis analysis.

For the rotor configuration, a two-stage procedure was adopted. First, a steady MRF calculation was performed for $N_{iter}=18,000$ iterations in order to obtain a stabilized initial flow field. The converged MRF solution was then used to initialize the transient sliding-mesh simulation. The rotor speed was set to ω=265 RPM, corresponding to a rotational period of T = 0.2264 s. Each revolution was divided into $N_{steps}=180$ physical time steps, leading to

(24) $$\Delta t=\frac{T}{180}=\frac{60}{265\times 180}\approx 0.00126\;s.$$

This time-step size corresponds to an angular advancement of Δφ=2° per step. The transient rotor simulation was advanced for $N_{rev}=10$ complete revolutions, resulting in $N_{steps}=1800$ physical time steps, with the final revolutions used for analysis after a periodic aerodynamic response was reached. The main numerical settings are summarized in Table 4.

### 3.4. Computational Resources, Modelling Assumptions, and Study Limitations

The computations were performed on a Dell Precision 3581 workstation equipped with a 13th-generation Intel Core i7-13800H processor operating at 2.50 GHz and 64 GB of RAM. ANSYS Fluent 2022 R1 was run in 2D, double-precision mode using 12 parallel solver processes; no GPU resources were assigned to the flow solver. The graphics adapter was therefore used only for visualization and post-processing. The computational times reported below are estimated wall-clock durations for the final medium-mesh cases and exclude geometry preparation, mesh generation, and post-processing.

A complete pitching-airfoil case required approximately 8 h. For the four-blade rotor configuration, each operating condition was obtained through a two-stage procedure: the steady MRF initialization required approximately 12 h, followed by a further 12 h for the transient Sliding Mesh calculation. The total runtime for one complete rotor case was therefore approximately 24 h. With 12 parallel solver processes, these durations correspond to estimated computational costs of approximately 96 core-hours for one pitching-airfoil case, 144 core-hours for each rotor stage, and 288 core-hours for one complete MRF-plus-Sliding-Mesh rotor calculation. Small variations in runtime occurred because the convergence rate and data-writing frequency were not identical for every operating condition.

The applicability of the results is nevertheless bounded by the modelling assumptions. Both the pitching-airfoil problem and the four-blade rotor representation were formulated at section level within a 2D framework. Consequently, spanwise transport, radial redistribution of loading, tip-vortex roll-up, helical wake development, 3D vortex stretching, and blade-vortex interaction were not resolved directly. The rotor calculation should therefore be interpreted as an unsteady analysis of periodic sectional loading and inter-section aerodynamic interaction, rather than as a complete 3D prediction of the full rotor wake.

The blade model also excludes spanwise twist and taper, structural flexibility, elastic deformation, and aeroelastic coupling. The trailing-edge-flap motion was prescribed kinematically, while actuator mass, hinge moments, transmission losses, actuation power, fatigue constraints, structural reinforcement, and installation-volume requirements were outside the scope of the aerodynamic simulations. In addition, the URANS formulation may diffuse vortex cores and smooth short-scale fluctuations during deep stall. Future work should therefore combine 3D rotor modelling with higher-fidelity turbulence treatment, structural dynamics, and actuator-integration analysis to quantify the influence of these effects on the predicted loads and control authority.

## 4. Results and Discussion

Post-processing was performed in MATLAB R2026 Software using the aerodynamic coefficients extracted from ANSYS Fluent 2022 R1. For the pitching-airfoil case, the final stabilized cycle was used to construct the aerodynamic hysteresis loops. For the rotor simulations, the final stabilized revolution was used to evaluate the periodic aerodynamic response under hover conditions. Flow-field contours were extracted at selected azimuthal positions to assess vortex formation, wake evolution, and unsteady aerodynamic effects.

### 4.1. Pitching Airfoil Results

**Figure 11:**
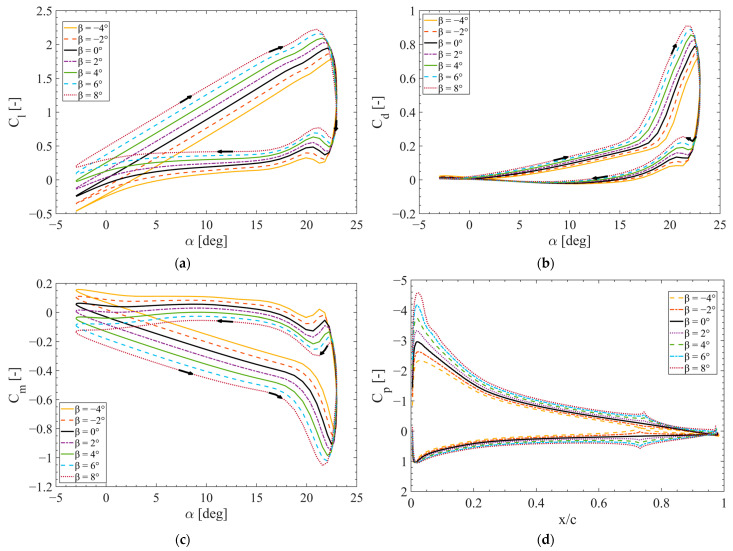
Comparison of the aerodynamic response between the baseline and TEF airfoils under dynamic stall, with arrows indicating the upstroke and downstroke directions: (**a**) $C_{l}$ variation; (**b**) $C_{d}$ variation; (**c**) $C_{m}$ variation; (**d**) $C_{p}$ distribution.

Figure 11 provides a coupled interpretation of the coefficient level hysteresis and the corresponding pressure field response induced by TEF deflection. As β increases from β=−4° to β=8°, the aerodynamic loading is amplified, indicating that positive TEF settings increase the induced aft-camber effect and strengthen the low-pressure loading during the pitching cycle. This trend is reflected in the $C_{l}-\alpha$ loop, where the positive β curves shift toward higher lift levels over both the increasing AoA and recovery branches. However, this lift augmentation is accompanied by a clear enlargement of the $C_{d}$ and $C_{m}$ loops near maximum AoA, indicating increased dissipative losses and torsional loading due to DSV growth, separated flow convection, and pressure centre migration.

The $C_{p}$ distribution explains this behaviour: β=8° generates the strongest leading-edge suction and aft pressure modification, whereas negative deflections weaken suction and unload the section. The TEF therefore reorganizes the chordwise pressure field, rather than acting only locally near the trailing edge. The separated upstroke and downstroke branches confirm a non-quasi-steady response governed by vortex formation, shedding, and wake recovery. Thus, positive β enhances lift but increases drag and moment penalties, while negative β reduces the hysteresis envelope and attenuates vortex-induced torsional loading.

#### 4.1.1. Influence of the Minimum TEF Deflection Angle on Unsteady Aerodynamic Loads

**Figure 12:**
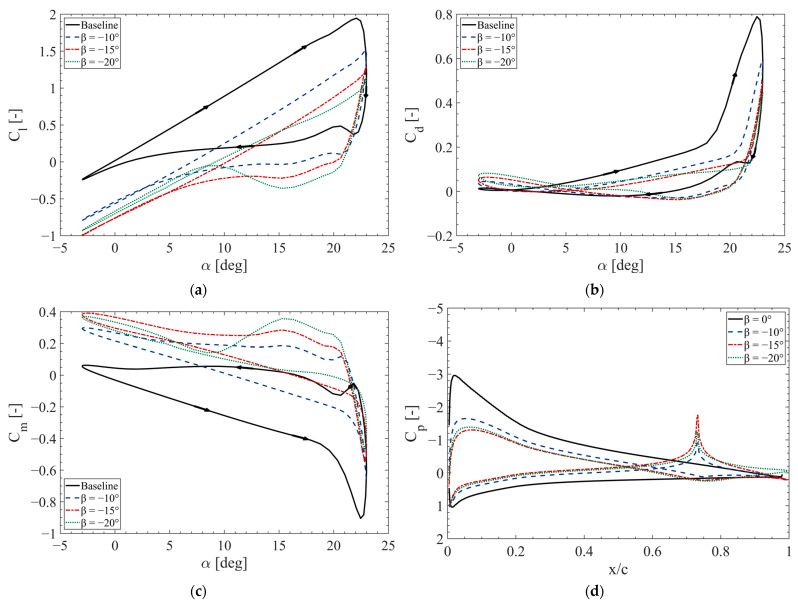
Aerodynamic response of the baseline and TEF airfoils for minimum TEF deflection angles, with arrows indicating the upstroke and downstroke directions: (**a**) $C_{l}$ variation; (**b**) $C_{d}$ variation; (**c**) $C_{m}$ variation; (**d**) $C_{p}$ distribution.

Figure 12 shows that increasingly negative TEF deflections progressively unload the airfoil across the dynamic stall evolution. Compared with the baseline, the negative β cases reduce the lift build-up and narrow the hysteresis envelope, with β=−15° and β=−20° showing the clearest attenuation of the DSV-induced overshoot in the high AoA region. Similar reductions are observed in $C_{d}$ and $C_{m}$, where the peak values decrease substantially, indicating weaker pressure centre migration and lower torsional loading throughout the stalled-flow stage of the dynamic-stall process. The $C_{p}$ distributions support this mechanism by revealing an attenuated leading-edge pressure deficit and a modified aft pressure recovery near the TEF region. Thus, negative β acts as a load-alleviation setting, reducing $C_{d}$ and $C_{m}$ penalties as well as hysteresis intensity, while requiring a trade-off with maximum $C_{l}$ capability.

#### 4.1.2. Influence of TEF Relative Chord Length on Unsteady Aerodynamic Loads

**Figure 13:**
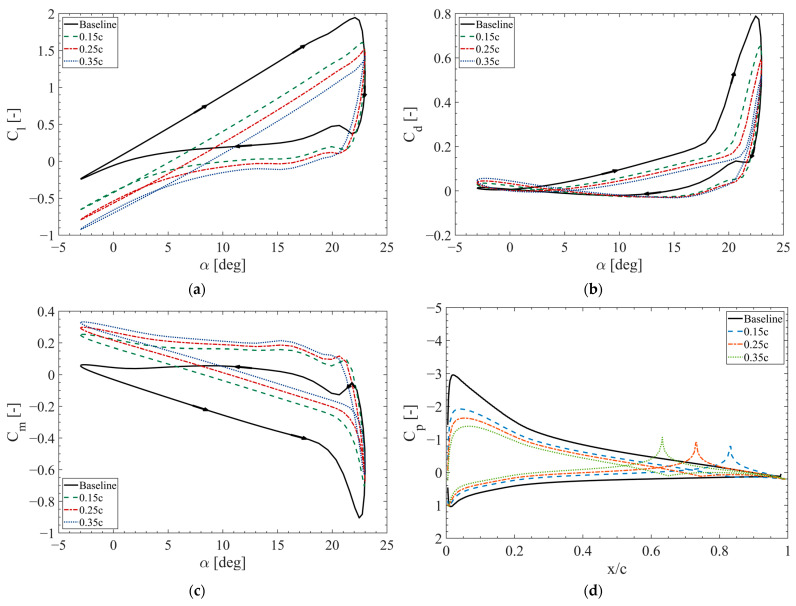
Aerodynamic coefficients for different relative TEF chord lengths, with arrows indicating the upstroke and downstroke directions: (**a**) variation of $C_{l}$; (**b**) variation of $C_{d}$; (**c**) variation of $C_{m}$; (**d**) distribution of $C_{p}$.

Figure 13 compares the baseline airfoil with TEF chord fractions of $c_{f}=0.15c$, $c_{f}=0.25c$, and $c_{f}=0.35c$, showing that increasing the actuated chord progressively changes the balance between lift preservation and dynamic stall load-alleviation. The baseline retains the largest $C_{l}$ peak and the widest hysteresis envelope, while the TEF cases reduce the lift build up and modify the recovery branch after the maximum AoA. Among the modified configurations, $c_{f}=0.15c$ preserves the highest lift level but provides the weakest reduction in the high AoA $C_{d}$ and $C_{m}$ peaks. By contrast, $c_{f}=0.35c$ produces the strongest attenuation of drag, negative pitching-moment peaks, and hysteresis intensity, indicating a more effective weakening of pressure centre migration induced by the DSV. However, this benefit is accompanied by the largest reduction in lift capability. The $C_{p}$ distributions support this interpretation by showing that longer TEF fractions more strongly reshape the aft pressure recovery and reduce the suction imbalance that feeds the separated flow structure. The selection of $c_{f}=0.25c$ was based on a multi-objective assessment that considered aerodynamic performance together with practical integration constraints. This intermediate configuration provides sufficient control authority to mitigate the main dynamic-stall penalties without producing the excessive aerodynamic unloading associated with the longest TEF. At the same time, the selected TEF chord length could reduce the aft-chord extent requiring structural modification, thereby facilitating actuator integration in future practical implementations.

#### 4.1.3. Flow Topology and Mach Number Contours for Different TEF Chord Fractions

**Figure 14:**
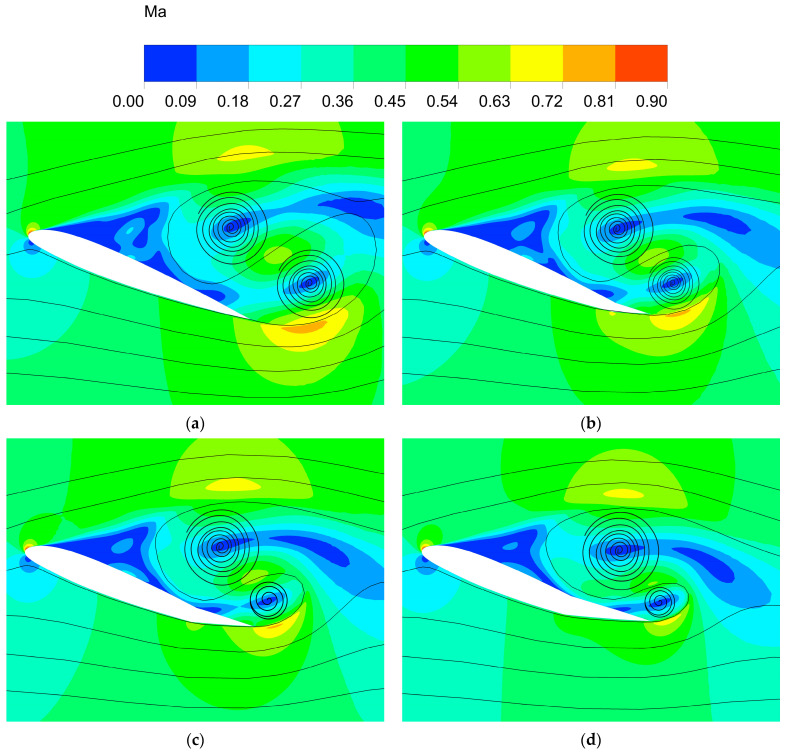
Streamlines and velocity contours at high AoA for the baseline airfoil and TEF chord-fraction configurations: (**a**) baseline; (**b**) $c_{f}=0.15c$; (**c**) $c_{f}=0.25c$; (**d**) $c_{f}=0.35c$.

The high AoA flow topology further reveals how $c_{f}$ governs DSV development and wake evolution (see Figure 14). In the baseline case, the aerodynamic response is governed by a large coherent DSV over the suction side and a secondary aft vortex, both associated with an extended low Mach wake and strong rear chord recirculation. Introducing a TEF progressively reorganizes this vortex system by altering the aft pressure recovery. For $c_{f}=0.15c$, the flow remains close to the baseline, indicating only limited influence on the separated shear layer. For $c_{f}=0.25c$, the aft vortex becomes more compact and shifts toward the TEF region, while the main DSV is moderated without excessively suppressing the aerodynamic loading. The $c_{f}=0.35c$ case produces the strongest wake smoothing and vortex contraction, confirming its greater load-alleviation potential but also explaining the larger lift reduction observed in the coefficient loops. Therefore, the contours support $c_{f}=0.25c$ as the most balanced TEF length, since it attenuates vortex-induced unsteadiness more effectively than $c_{f}=0.15c$ while avoiding the excessive unloading associated with $c_{f}=0.25c$.

The following procedure is applied separately to the main DSV and the secondary DSV. All quantities must be extracted at the same flow time, AoA, and pitching branch for every configuration (see Table 5). For the current comparison, the selected state is t = 0.3906 s and α=21.809° on the downstroke.

For each isolated vortex region $A_{v}$, the dimensional circulation was evaluated from the out-of-plane vorticity component:

(25) $$\Gamma_{v}\;=\iint_{A_{v}}\omega_{z}dA\;,$$

where $\omega_{z}$ is the vorticity component normal to the 2D computational plane. Since the circulation sign depends on the direction of rotation, the signed value was retained in the raw data, whereas its magnitude was used for comparing vortex strength. The non-dimensional circulation was defined as follows:

(26) $$\Gamma_{v}^{*}\;=\frac{\left|\Gamma_{v}\right|}{U_{\infty }c}\;.$$

The peak vortex intensity was characterized by the maximum magnitude of the out-of-plane vorticity within the same isolated region:

(27) $$\omega_{z,max}\;=\underset{A_{v}}{\max}\left|\omega_{z}\right|.$$

Its non-dimensional form was calculated as follows:

(28) $$\omega_{z,max}^{*}\;=\frac{\omega_{z,max}c}{U_{\infty }}.$$

The size of each vortex was described by its enclosed area,

(29) $$A_{v}=\iint_{A_{v}}dA,$$

which was normalized using the chord length:

(30) $$A_{v}^{*}\;=\frac{A_{v}}{c^{2}}.$$

The quantitative data support the visual trends while providing a clearer distinction between the vortex structures. Relative to the baseline, $c_{f}=0.25c$ reduces the main DSV circulation and area by approximately 13.7% and 20.8%, respectively, while the secondary-vortex area decreases by about 32.4%. The $c_{f}=0.35c$ case produces the strongest attenuation of the secondary structure, with reductions of approximately 46.0% in circulation and 70.0% in area, although the main DSV circulation increases slightly by about 4.6%. Thus, $c_{f}=0.25c$ provides the most balanced reduction of both primary and secondary vortex activity.

### 4.2. Four-Blade Rotor with TEF Results

**Figure 15:**
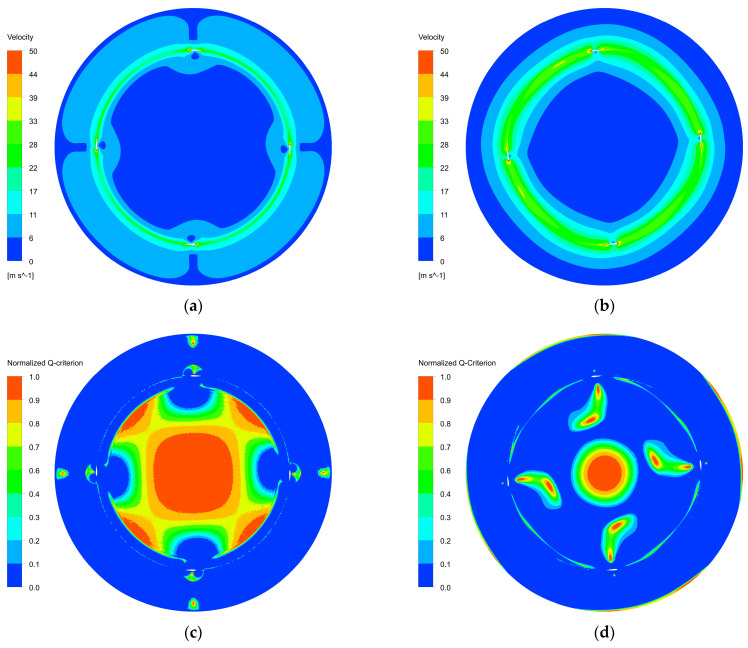
Velocity contours and Q-criterion structures for the four-section rotor configuration: (**a**) MRF velocity field; (**b**) Sliding Mesh velocity field; (**c**) MRF Q-criterion; (**d**) Sliding Mesh Q-criterion.

For the baseline rotor configuration, the comparison between the MRF and Sliding Mesh approaches highlights the limitations of the steady rotating-frame approximation in representing the unsteady rotor flow (see Figure 15). Although the MRF provides a relatively uniform mean-flow field suitable for initialization, the Sliding Mesh method captures the instantaneous section motion, wake redistribution, blade-passage effects, and vortex interactions.

The quantitative comparison in Table 6 shows differences between the two approaches, with relative differences of approximately 134% and 99% for the mean $C_{l}$ and $C_{d}$ of Section 1, respectively, and about 91% for the mean rotor $C_{m}$. These discrepancies confirm that MRF is appropriate mainly for initialization and preliminary flow estimation, whereas Sliding Mesh is required for the final assessment of unsteady aerodynamic loads and rotor moment.

The imposed UDF defines a collective pitching law, ensuring that all four blade sections experience the same instantaneous AoA variation during each rotor revolution. Therefore, the coefficients extracted for Section 1 can be considered representative of the identical blade sections, while $C_{m}$ describes the combined rotor response of the four simultaneously actuated sections.

**Figure 16:**
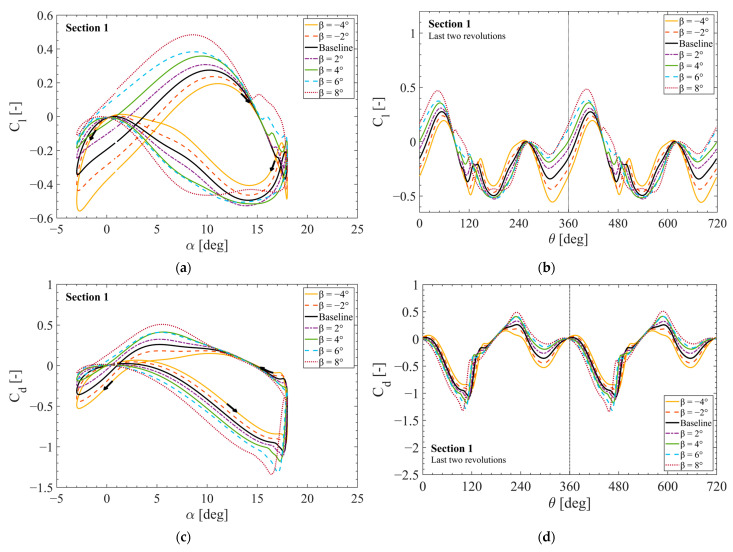
Aerodynamic response of the rotor configuration with collective TEF actuation, with arrows indicating the direction of the aerodynamic hysteresis loops: (**a**) Section 1, $C_{l}$ hysteresis; (**b**) Section 1, $C_{l}$ over the last two revolutions; (**c**) Section 1, $C_{d}$ hysteresis; (**d**) Section 1, $C_{d}$ over the last two revolutions.

Figure 16a shows that TEF actuation modifies both the lift level and the hysteretic structure of the dynamic stall response. The separated upstroke and downstroke branches confirm an unsteady response governed by delayed separation, DSV formation, vortex convection, and wake recovery. Positive β increases the upper branch, especially at moderate and high AoA, due to stronger aft camber effects and enhanced suction side loading. At higher positive β, the secondary loop observed for the baseline and lower β cases is strongly reduced, indicating attenuation of DSV related lift fluctuations and wake interaction. Conversely, negative β unloads the section and acts as a load-alleviation setting.

The azimuthal lift history in Figure 16b confirms that the final revolutions reached a periodic unsteady state. Although the imposed motion is sinusoidal, the lift waveform contains secondary peaks and troughs generated by nonlinear separated flow effects. The curve ordering remains consistent with the hysteresis plot, with positive β producing the highest lift peaks, the baseline remaining intermediate, and negative β reducing sectional loading. Figure 16c,d show the aerodynamic cost of lift enhancement: positive TEF settings enlarge the drag envelope, particularly at high AoA, whereas negative β reduces drag amplitude and weakens the dissipative effects associated with dynamic stall.

**Figure 17:**
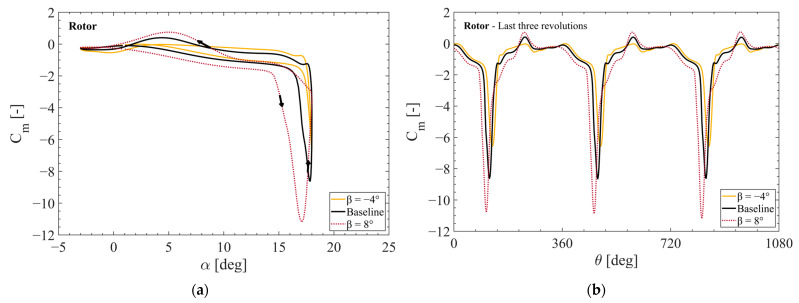
Aerodynamic response of the rotor configuration with collective TEF actuation, with arrows indicating the direction of the hysteresis loop: (**a**) rotor $C_{m}$ hysteresis; (**b**) rotor $C_{m}$ over the last three revolutions.

The $C_{m}$ plots in Figure 17a,b are important because they describe the rotor level response of the four simultaneously actuated sections. The sharp negative drop near maximum AoA is characteristic of dynamic stall and moment stall and results from the aft migration of the pressure centre as the DSV convects toward the trailing edge. While the baseline shows a marked negative peak, β=8° amplifies this torsional penalty due to the additional lift generated by positive TEF deflection. In contrast, β=4° reduces the moment drop and promotes smoother recovery, indicating a load-alleviation effect. This negative peak repeats periodically at maximum collective incidence.

Overall, TEF morphing controls the rotor aerodynamic response through camber-induced lift modulation and vortex-induced load regulation: positive β, especially β=6° and β=8°, enhances lift and partially suppresses the secondary high AoA loop, but increases drag and pitching moment penalties, whereas negative β reduces hysteresis, weakens dynamic-stall-induced drag and moment peaks, and smooths load evolution at the cost of lift capability. Thus, TEF morphing provides a controllable trade-off between lift enhancement, DSV mitigation, and unsteady load reduction under hover conditions.

**Figure 18:**
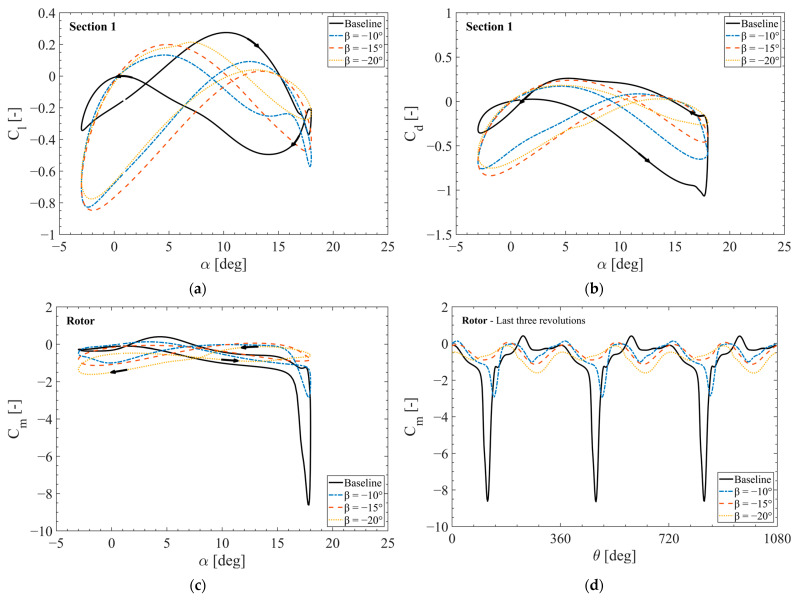
Aerodynamic coefficients obtained for the rotor configuration with minimum TEF deflection angles, with arrows indicating the direction of the hysteresis loop: (**a**) Section 1, $C_{l}$ versus AoA; (**b**) Section 1, $C_{d}$ versus AoA; (**c**) rotor, $C_{m}$ versus AoA; (**d**) rotor pitching, $C_{m}$ over the last three revolutions.

Figure 18 extends the rotor analysis to larger negative TEF deflections, β=−10°, β=−15°, and β=−20°, compared with the baseline configuration. The $C_{l}-\alpha$ loop shows that the baseline develops a wide dynamic-stall hysteresis, associated with delayed separation, DSV formation, and slow post-stall recovery. Negative TEF deflections smooth this response and reduce the abrupt high-AoA lift variation, especially for β=−15° and β=−20°, indicating weaker separated-flow development and partial suppression of the DSV-induced overshoot. The same tendency appears in the drag response, where the negative TEF cases reduce the hysteresis envelope and promote a more gradual transition toward stalled flow.

The main improvement is observed in the rotor moment coefficient. The baseline exhibits a severe negative moment drop near maximum incidence, caused by pressure-centre migration during DSV convection. This moment-stall event is reduced by negative TEF deflection: β=−10° still preserves a residual drop, while β=−15° and β=−20° strongly suppress the abrupt collapse and smooth the periodic torsional loading over the final revolutions. Overall, larger negative TEF deflections act as dynamic-stall load-alleviation settings, reducing separation effects, DSV-related hysteresis, and severe moment excursions. However, β=−10° preserves more lift but provides weaker control, whereas β=−20° gives the strongest suppression but greater unloading. Thus, β=−15° offers the most balanced compromise between drag and moment reduction and useful aerodynamic loading.

**Figure 19:**
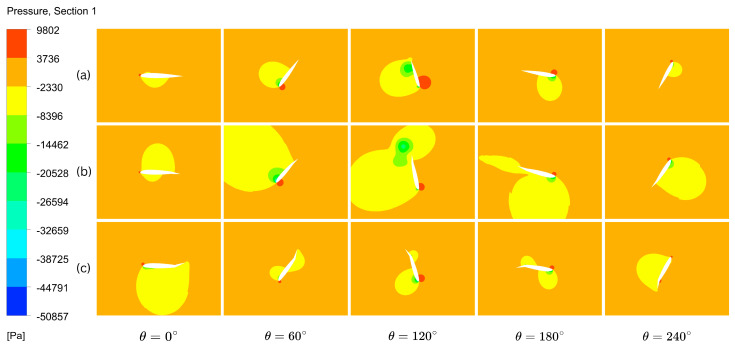
Pressure contours around Section 1 at selected azimuthal positions for: (**a**) baseline airfoil; (**b**) TEF deflection, β=8°; (**c**) TEF deflection, β=−20°.

The pressure contours presented in Figure 19 indicate that the baseline configuration develops an azimuth-dependent loading pattern. At θ=0°, the pressure field remains compact, corresponding to a moderate incidence state, whereas at θ=60° and especially θ=120°, the suction-side pressure deficit intensifies and the aft pressure concentration increases. This evolution suggests an adverse pressure gradient, boundary layer destabilization, and DSV convection over the chord. The residual aft suction at θ=180° and its weakening by θ=240° indicate wake memory effects during recovery.

For β=8°, the contours show stronger suction side loading than in the baseline case, particularly at θ=60°, θ=120°, and θ=180°. The increased effective aft camber intensifies rear chord pressure demand and enhances lift generation, while the enlarged low-pressure regions indicate stronger pressure centre migration during DSV formation and convection. Thus, β=8° acts mainly as a lift enhancement setting, with possible drag and pitching moment penalties under high AoA separated flow conditions.

By contrast, β=−20° produces a more compact and less intense pressure field. Although suction remains visible at θ=60° and θ=120°, it is more localized and lacks the extended pressure deficit observed for β=8°. This confirms that large negative TEF deflection unloads the section, modifies aft pressure recovery, weakens DSV growth, and supports smoother post-stall recovery. Overall, TEF morphing modifies the entire chord wise pressure topology: positive β enhances lift and may intensify dynamic stall effects, whereas negative β attenuates vortex induced pressure migration and promotes load-alleviation.

**Figure 20:**
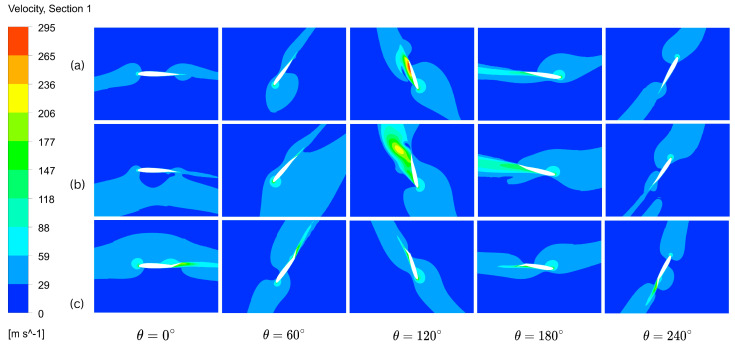
Velocity contours around Section 1 at selected azimuthal positions for: (**a**) baseline airfoil; (**b**) TEF deflection, β=8°; (**c**) TEF deflection, β=−20°.

The velocity contours presented in Figure 20 complement the pressure-field analysis by showing the acceleration zones, wake deficits, and shear-layer structures around the rotor Section 1. In the baseline case, a strong suction-side high-velocity region develops at θ=120°, indicating shear-layer acceleration, separation onset, DSV formation, and a pronounced wake deficit, while the disturbed flow at θ=180° and θ=240° confirms wake-memory effects. For β = 8°, the suction-side and aft-chord acceleration becomes stronger and more extended, confirming enhanced loading and lift production, but also higher shear-layer activity and possible drag and moment penalties. By contrast, β=−20° produces narrower acceleration zones, a smoother wake footprint, weaker DSV development, and flow recovery.

The present trends agree with international experimental and numerical studies showing that an active TEF modifies dynamic stall mainly through aft chord pressure recovery, vortex convection, and the associated pitching moment response [18,19,21,41]. Feszty et al. [18] related moment alleviation to changes in aft chord vortex dynamics, while Gerontakos and Lee [19] demonstrated that flap deflection and timing can substantially reshape the unsteady load loops. Raiola et al. [41] and Xing et al. [21] similarly reported that active flap motion can attenuate dynamic stall loads without necessarily suppressing DSV formation. Consistent with these findings, the present positive deflections enhanced lift but increased drag and negative pitching moment peaks, whereas negative deflections narrowed the hysteresis loops and reduced the separated flow intensity.

Recent morphing-airfoil studies likewise emphasize that control effectiveness depends on deformation amplitude, phase, and the chordwise extent of the morphing region [21,42]. The present contribution adds evidence for the NACA 13112 and for a rotorcraft-representative operating condition, showing that the TEF chord fraction governs a measurable trade-off between load-control authority and aerodynamic unloading.

The MRF–Sliding Mesh comparison is also consistent with the broader interpretation of steady and transient rotating-domain methods. A steady rotating frame can reproduce a mean-flow estimate but necessarily filters the periodic motion, wake memory, and load modulation captured by a time-resolved mesh. The unusually large differences found here also indicate that consistent reference values, force directions, and moment centres must be verified before attributing the entire discrepancy to physical modelling alone.

Reliable rotor-load assessment requires not only the prediction of aerodynamic forces but also their quantitative characterization under transient conditions. Recent studies have proposed regularization-based and data-efficient approaches for identifying harmonic and nonlinear dynamic loads from structural responses [43,44]. Although these methods address inverse load reconstruction rather than direct aerodynamic prediction, they emphasize the importance of explicit error metrics and robust load evaluation. In the present study, this requirement is addressed through the quantitative comparison of the Section 1 aerodynamic coefficients and the total rotor pitching-moment coefficient obtained using the MRF and Sliding Mesh approaches.

For a helicopter rotor, the TEF can be interpreted as an adaptive aft-camber device activated only during the critical part of the azimuthal or pitching cycle. A negative deflection during DSV growth and moment stall could reduce drag, torsional loading, and pressure-centre migration; the TEF could then return to a neutral or mildly positive setting when lift recovery is required. This phase-scheduled strategy follows the biomimetic principle of changing shape only when the aerodynamic demand justifies it.

Potential machine-level benefits include reduced pitch-link and torsional loads, attenuation of vibratory loading, improved transient lift control, and a possible reduction in fatigue accumulation. However, aerodynamic benefit alone does not guarantee a positive system-level outcome. Practical integration requires sufficient actuator bandwidth and authority, low added mass, limited hinge or internal actuation loads, fatigue-resistant compliant skins, structural reinforcement, transmission efficiency, fail-safe locking, and adequate internal blade volume.

The current results therefore provide an aerodynamic design envelope rather than a flight-ready solution. The next stage should combine 3D rotor CFD, aeroelastic coupling, actuator and compliant-structure models, and experimental blade-segment testing. The same adaptive-camber principle may also be transferable to wind-turbine blades, marine and tidal rotors, propellers, and manoeuvrable UAVs exposed to cyclic separation and rapidly varying loads.

## 5. Conclusions

This study applied an established CFD toolchain to quantify the effects of a TEF on dynamic stall over a pitching NACA 13112 airfoil and on a four-section configuration representative of the IAR 330 PUMA main rotor. The framework combined UDF-prescribed motion, transient URANS calculations, aerodynamic-coefficient analysis, and pressure- and velocity-field interpretation, enabling a consistent assessment at both airfoil and rotor-section levels.

The pitching-airfoil results confirmed a strongly non-quasi-steady aerodynamic response, characterized by distinct upstroke and downstroke branches in $C_{l}$, $C_{d}$, and $C_{m}$. Positive TEF deflections increased aft camber and sectional lift, particularly for β=6° and β=8°, but also increased drag and intensified the negative pitching-moment peak at high AoA. Negative deflections narrowed the hysteresis loops and reduced drag and moment penalties, although at the cost of lower maximum lift.

The chord-fraction analysis revealed a clear aerodynamic compromise among the three tested geometries. The $c_{f}=0.15c$ configuration preserved the highest lift, whereas $c_{f}=0.35c$ produced the strongest unloading and vortex attenuation. The intermediate $c_{f}=0.25c$ configuration provided the most balanced response and reduced the peak $C_{m}$ by approximately 32% relative to the baseline under the investigated conditions. This result supports its selection as the reference configuration for future structural and actuator integration.

The pressure and velocity fields confirmed that the observed load changes were associated with modified aft-chord pressure recovery, shear-layer roll-up, DSV convection, and wake development. At rotor level, these mechanisms remained identifiable despite blade-passage and wake-interaction effects. The largest negative deflections, particularly β=−15° and β=−20°, produced the strongest smoothing of the load histories and the clearest attenuation of the moment variations associated with pressure-centre migration.

The quantitative MRF–Sliding Mesh comparison yielded relative differences of approximately 134% in the mean Section 1 $C_{l}$, 99% in the mean Section 1 $C_{d}$, and 91% in the mean rotor $C_{m}$. These results show that the steady rotating-frame approximation is suitable mainly for initialization and preliminary mean-flow estimation, whereas Sliding Mesh is required for resolving instantaneous section motion, wake redistribution, blade-passage effects, and unsteady rotor loads.

Overall, TEF morphing can be scheduled either for lift enhancement or for load alleviation, and no single deflection is optimal for all operating states. The engineering relevance of the biomimetic concept lies in localized and reversible camber adaptation, allowing the rotor section to change shape only when required by the aerodynamic loading. Future work should combine 3D rotor CFD, aeroelastic coupling, structural and actuator constraints, and phase-controlled or azimuth-dependent TEF actuation to quantify the resulting power, vibration, fatigue, and control benefits at machine level.

## Figures and Tables

**Table 1:** Geometric configurations of the baseline and TEF-deflected airfoil at different AoA and β.

AoA	*β*	Original Airfoil	Airfoil with TEF
$0^{{}^{\circ}}$	$0^{{}^{\circ}}$	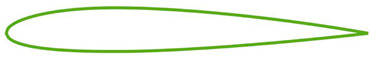	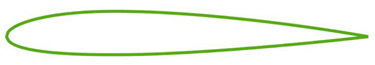
$10^{{}^{\circ}}$	$-10^{{}^{\circ}}$	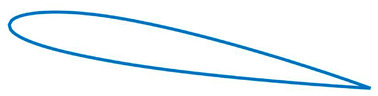	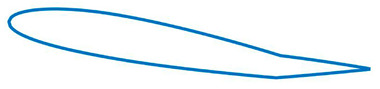
$15^{{}^{\circ}}$	$-15^{{}^{\circ}}$	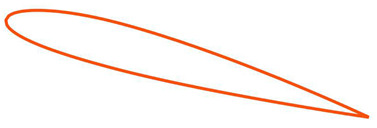	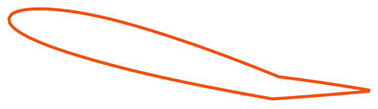
$-3^{{}^{\circ}}$	$8^{{}^{\circ}}$	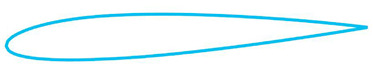	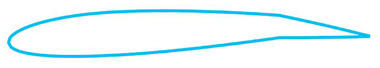
$0^{{}^{\circ}}$	$8^{{}^{\circ}}$	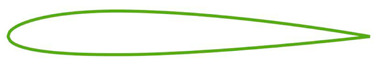	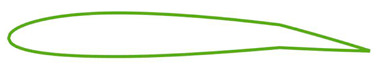

**Table 2:** Computational mesh parameters used for the pitching-airfoil grid-sensitivity study.

Mesh Control	Mesh Parameter	Coarse Mesh	Medium Mesh	Fine Mesh
Body Sizing	Airfoil domain size [mm]	2.5	1.5	1
	Far-field domain size [mm]	40	25	20
Edge Sizing	Airfoil edge size [mm]	1.5	1.0	0.5
Inflation	First-layer height [mm]	$6.40\times 10^{-3}$	$3.20\times 10^{-3}$	$1.60\times 10^{-3}$
	Number of inflation layers	40	50	60
	Inflation growth rate	1.18	1.15	1.12
Elements Number	Total number of cells	$6.39\times 10^{5}$	$1.69\times 10^{6}$	$3.40\times 10^{6}$

**Table 4:** Computational framework and numerical methodology.

Parameter	Value/Setting
Turbulence model	SST k-ω
Solver	Pressure-based
Numerical approach	Steady MRF/Transient Sliding Mesh
Rotor speed	265 RPM
Mach number	M≈0.43
Air Density	Ideal gas
Air Viscosity	$1.7894\times 10^{-5}\;\mathrm{Pa}\cdot s$
Turbulent Intensity	5%
Tip Speed Ratio	1
Calculation algorithm	Coupled
Pressure–velocity coupling	Coupled
Spatial discretization schemes	Second Order
Pressure interpolation	Second Order
Density equation	Second Order Upwind
Momentum equation	Second Order Upwind
Turbulent kinetic energy equation	Second Order Upwind
Specific Dissipation Rate	Second Order Upwind
Energy	Second-order upwind
Transient formulation	First-Order implicit
Maximum residuals	Maximum residuals $1\times 10^{-6}$

**Table 5:** Quantitative characteristics of the main and secondary DSVs for the baseline and TEF chord-fraction configurations at α=21.81° on the downstroke.

Configuration	Vortex Structure	$\Gamma_{v}^{*}$	$\omega_{z,max}^{*}$	$A_{v}^{*}$
Baseline	Main DSV	0.981085	15.2005	0.098617
	Secondary vortex	0.919045	4.5262	0.0696239
$c_{f}=0.15c$	Main DSV	0.870731	15.9566	0.0810919
	Secondary vortex	0.987847	1.72321	0.0593121
$c_{f}=0.25c$	Main DSV	0.847116	16.7726	0.0781171
	Secondary vortex	0.918644	2.45906	0.0470473
$c_{f}=0.35c$	Main DSV	1.02587	16.1584	0.0965997
	Secondary vortex	0.496178	3.94128	0.0208933

**Table 6:** MRF–Sliding Mesh quantitative comparison of Section 1 loads and rotor pitching moment.

Aerodynamic Parameter	MRF	Sliding Mesh	Relative Difference (%)
Mean Section 1 $C_{l}$	−0.3754	−0.16047	133.95%
Mean Section 1 $C_{d}$	−0.00301	−0.22107	98.64%
Mean rotor $C_{m}$	−0.09313	−1.06573	91.26%

## Data Availability

The original contributions presented in this study are included in the article, further inquiries can be directed to the corresponding authors.
